# Supplementary material for: A microRNA Profile Regulates Inflammation-Related Signaling Pathways in Young Women with Locally Advanced Cervical Cancer
Source: Cells. 2024 May 23;13(11):896. doi: 10.3390/cells13110896 (PMC11172105; doi:10.3390/cells13110896)
Supplement: Supplementary file 1 [file cells-13-00896-s001.zip › Supplementary table 1.docx]

Supplementary table 1. List of primers used for mRNA expression

| **Gene** | **Primer sequence 5´- 3´** |
| --- | --- |
| β-Actin | Fw ATGACTTAGTTGCGTTACACCCT |
|  | Rv TGCTCGCTCCAACCGACTG |
| JAK1 | Fw TGGATTACAAGGATGACGAAGGAA |
|  | Rv CGGACACAGACGCCATAGAG |
| STAT3 | Fw CAGCAGCTTGACACACGGTA |
|  | Rv AAACACCAAAGTGGCATGTGA |
| CCND1 | Fw TTG CAA GCA GGA CTT TGA GGC AAG |
|  | Rv CAA ACA CCA GTT GGC ACC AAA GGA |
| CXCL10 | Fw TGT ACG CTG TAC CTG CAT CAG |
|  | Rv TGA TGG CCT TCG ATT CTG GAT |
