# Supplementary material for: A microRNA Profile Regulates Inflammation-Related Signaling Pathways in Young Women with Locally Advanced Cervical Cancer
Source: Cells. 2024 May 23;13(11):896. doi: 10.3390/cells13110896 (PMC11172105; doi:10.3390/cells13110896)
Supplement: Supplementary file 1 [file cells-13-00896-s001.zip › Suplemmentary table 3.docx]

| **Gene ID** | **Fold change** | **q-value** |
| --- | --- | --- |
| ILR6 | 1.4651 | 0.3313 |
| JAK1 | 7.3516 | 0.0006 |
| JAK2 | 10.5655 | 0.0006 |
| STAT3 | 9.3519 | 0.0006 |
| STAT5B | 6.9242 | 0.0006 |

Supplementary Table S3. Genes of the JAK-STAT signaling pathway overexpressed in a cohort of Mexican patients with LACC (Gene Expression Omnibus with the accession number GSE56303, http://www.ncbi.nlm.nih.gov/geo/).
