# Supplementary figures and images for: A microRNA Profile Regulates Inflammation-Related Signaling Pathways in Young Women with Locally Advanced Cervical Cancer
Source: Cells. 2024 May 23;13(11):896. doi: 10.3390/cells13110896 (PMC11172105; doi:10.3390/cells13110896)

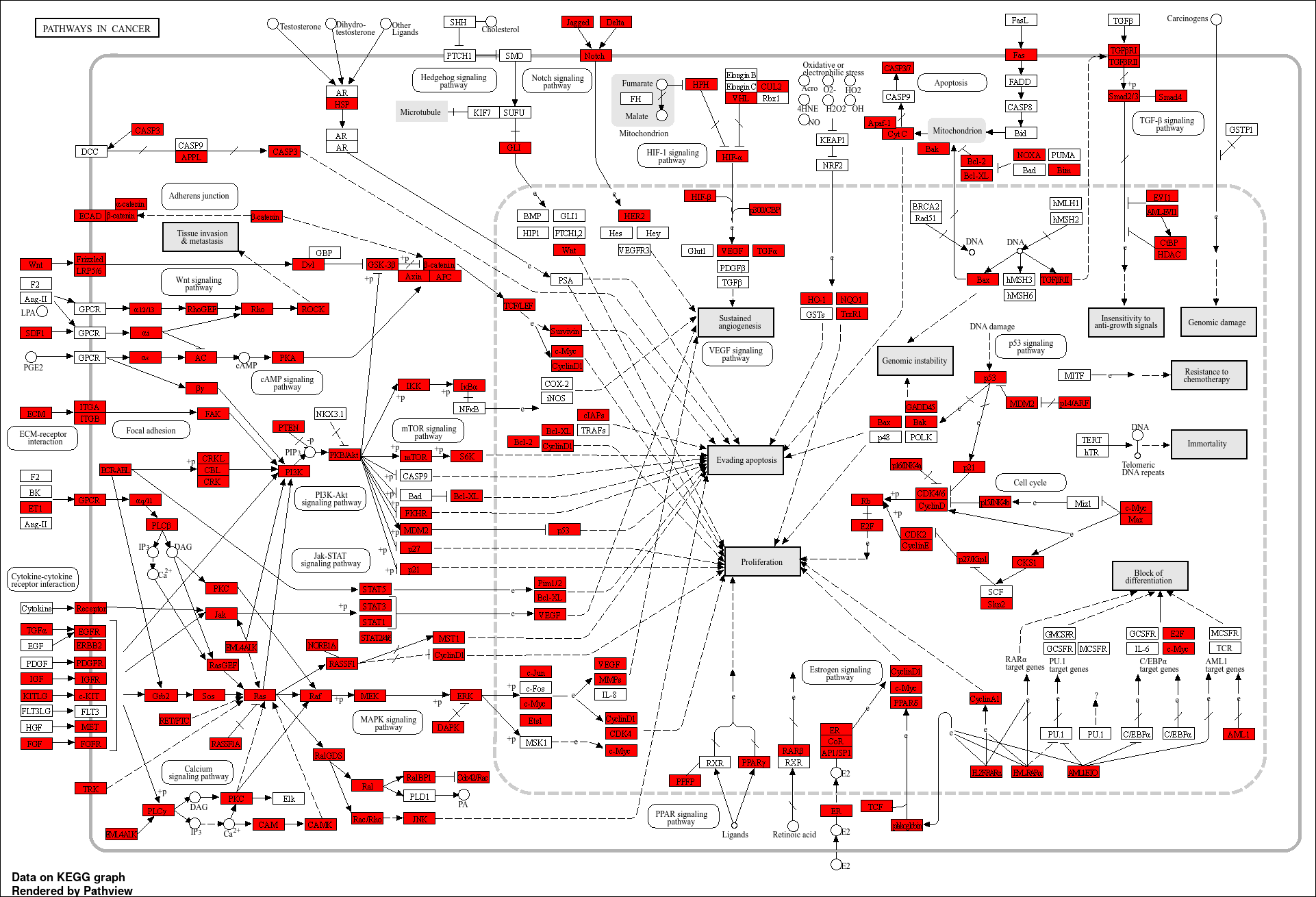

Supplement: Supplementary file 1 [file cells-13-00896-s001.zip › Supplementary figure 1.png]
